# The PSR corpus: A Persian sentence reading corpus of eye movements

**DOI:** 10.3758/s13428-024-02517-x

**Published:** 2024-12-11

**Authors:** Zohre Soleymani Tekbudak, Mehdi Purmohammad, Ayşegül Özkan, Cengiz Acartürk

**Affiliations:** 1 Cognitive Science Department, Orta Dogu Teknik Universitesi, Ankara, Türkiye; 2 Department of Education, University of Alberta, Edmonton, Canada; 3 Centre for Cognitive Science, Jagiellonian University, Ul. Ingardena 3, 304, 30-060 Kraków, Poland

**Keywords:** Eye movements, Oculomotor control in reading, Silent reading, Right-to-left script reading, Persian

## Abstract

The present study introduces the Persian Sentence Reading (PSR) Corpus, aiming to expand empirical data for Persian, an under-investigated language in research on oculomotor control in reading. Reading research has largely focused on Latin script languages with a left-to-right reading direction. However, languages with different reading directions, such as right-to-left and top-to-bottom, and particularly Persian script-based languages like Farsi and Dari, have remained understudied. This study pioneers in providing an eye movement dataset for reading Persian sentences, enabling further exploration of the influences of unique Persian characteristics on eye movement patterns during sentence reading. The core objective of the study is to provide data about how word characteristics impact eye movement patterns. The research also investigates the characteristics of the interplay between neighboring words and eye movements on them. By broadening the scope of reading research beyond commonly studied languages, the study aims to contribute to an interdisciplinary approach to reading research, exemplifying investigations through various theoretical and methodological perspectives.

## Introduction

Despite their complementary role in the processes that underlie reading, research on relevant fields such as sentence comprehension, grammatical processes, and text comprehension have remained distinct fields of research than letter and word recognition. Although the research on psycholinguistics has addressed the latter (e.g., Rastle, 2016; Norris, 2013), the integration of information across fixations remains an unresolved issue (Nikolaev & Van Leeuwen, 2019). Visual word recognition has been proposed to encompass multiple stages of processing, including morpho-phonological information retrieval at the lexeme level, accessing syntactic information at the lemma level, and accessing the lexical representation at the conceptual level (Levelt et al., 1999). From a methodological standpoint, most research in psycholinguistics relies on single-word methods, yielding robust findings on the processing of letters within words and, more broadly, on word recognition. These findings have paved the way for the development of early computational models of word recognition (e.g., Gaskell et al., 2007; Paap et al., 1982; Reichle, 2021). Nevertheless, the investigation of eye movements in reading represents a distinct yet complementary approach, one that assumes greater environmental validity but also brings with it the complexities of data analysis due to its focus on the integration of information across fixations during reading. Thus, reading research focuses on patterns of information intake in addition to assessing the patterns of lexical access or word recognition from a psycholinguistic perspective. As a result, oculomotor control in reading has emerged as an independent field of research. Complementing studies on psycholinguistics and cognitive development, it focuses on a specific problem: the processes of integrating information across fixations.

Over the past several decades, the evolution of reading research has largely occurred within the context of cognitive and developmental psychology (Pollatsek & Treiman, 2015; Snowling & Hulme, 2022). Stemming from an initial interest kindled long ago (Huey, 1908), researchers have employed eye tracking as a methodology to explore the allocation of visual attention during reading. The fundamental research question in oculomotor control within reading research stems from the relationship between word characteristics and fixation patterns: when and where to move the eyes. Eye movement recordings have served as a valuable tool for investigating the underlying processes of oculomotor control in reading. The timing of eye movements is evaluated through the analysis of fixation durations, while the determination of where to move the eyes is studied through the analysis of fixation locations and saccadic amplitudes during reading. The null hypothesis posits those variations in eye movement patterns—specifically, fixation durations and saccadic patterns—are random, thus being independent of the text stimuli. Although the prevalent assumption was that stimuli did not influence when and where to move the eyes in early research on reading, subsequent studies have uncovered systematic relationships between text stimuli characteristics and eye movement patterns (e.g., Rayner, 1998; Rayner, 2009; Schotter et al., 2012).

The central question explores how information flow and integration occur during text reading and how word characteristics influence eye movement patterns. Three major determinants identified by previous reading research are the length of a word, its frequency of daily use (typically measured by the number of occurrences in a text corpus), and the predictability of a word in a sentential context. These factors significantly impact several oculomotor variables—dependent variables in this case—particularly the first fixation on a word and both incoming and outgoing saccadic amplitudes (Kliegl, 2007; Rayner et al., 2012). These factors have been partially shown to have an impact on the integration of information across fixations on multiple words. The current research on oculomotor control in reading mostly focuses on the current fixation on a word (*n*), the previous fixation (*n − 1*), and the next fixation (*n* + *1*), and their relationship to specific features of the words, such as the frequency of daily use, length in terms of the number characters, and the predictability of a word in the context of a sentence (sentential predictability). The relationship between the processing of the words *n − 1*, *n*, and *n* + *1*, and the fixations on them have been investigated under the umbrella term *parafoveal information intake*. The term finds its roots as a functional specification of early studies on perceptual processes in reading by Huey (1908).

The interplay between neighboring words reflects the integration of information across fixations in reading. Numerous influences, notably those tied to the properties of the currently fixated word (*n*), as well as spillover frequency effects from the preceding word (*n − 1*), have been well established in the literature. However, the debate persists as recent research reveals divergent findings concerning the impact of these factors in proximity to the fixated word. Particularly contentious are influences related to the preview of the subsequent word (*n* + *1*) (Angele et al., 2015; Brothers et al., 2017; Kliegl, 2007; Kliegl et al., 2006; Özkan et al., 2021; Rayner et al., 2007). While both serial and parallel models predict *n* + *1* identical preview effects, the two models do not agree on the *semantic* preview effect. For example, Yan et al. (2009) reported significantly shorter gaze durations on pretarget words in the semantic preview condition compared to those in the unrelated previews. Accordingly, semantic preview effects might have been conceived more problematic for serial-attention-shift models (e.g., Reichle et al., 2009), but less so for parallel distributed processing models (e.g., Engbert et al., 2005). Rayner et al. (2003) stated that “the basis for the robust parafoveal preview benefit obtained in numerous studies is not any type of semantic code” (p. 230), highlighting that semantic preview effects would be problematic for serial-attention-shift models. However, later research has shown that semantic preview benefit effects are not necessarily incompatible with serial-attention-shift models. Consequently, these effects have been a significant research focus, often employed to evaluate theoretical frameworks. This is a key point of contention in the theory of eye movement control in reading, and its translation into computational models has been a major focus for the past two decades (see Radach et al., 2007; Reichle, 2021, for overviews of the theoretical debate).

We will not cover the literature on the preview benefit effect in its entirety here, as the present study does not contain a gaze-contingent display change manipulation. On the other hand, the debate reflects commonalities and differences in eye movement patterns across languages (see Schotter & Jia, 2016; Schotter et al., 2015, for English; Hohenstein & Kliegl, 2014, for German; Yan et al., 2012; Yang et al., 2014; Li et al., 2018, for Chinese). Given that traditional Chinese script permits texts to be inscribed both horizontally from right to left and vertically from top to bottom, it affords an opportunity to explore the impact of reading direction while controlling for the confounding variable of differential familiarity with reading orientations (Yan et al., 2019). For instance, Yan et al. (2024) examined how direction-specific reading experience affects the perceptual span. They recorded the eye movements of traditional Chinese readers as they read sentences presented both horizontally and vertically. The results demonstrated that optimal reading performance was achieved under a smaller window condition in vertical reading compared to horizontal reading, indicating a generally smaller perceptual span for the former. Another study is Zhou et al. (2021), which investigated the perceptual span of proficient Uighur readers during the natural reading of sentences. This study represents the inaugural documentation of the perceptual span within a horizontally leftward-oriented script. By comparing various window conditions to the baseline condition, the study found that Uighur readers achieved optimal performance in terms of reading speed and gaze duration when the viewing window exposed a minimum of five letters to the right and 12 letters to the left of the currently fixated letter.

In these investigations of commonalities and differences in eye movement patterns, corpora of eye-movement data have served as a foundation for reproducible analyses and testing of theoretical assumptions and oculomotor control models. Over the past two decades, the progress in computational models has demonstrated that systematic aspects of reading patterns can be simulated, drawing on empirical studies (Reichle, 2021). Eye movement datasets enrich the field by providing data that may aid in the development of computational models of reading and the diagnosis of reading disorders, such as dyslexia, in specific languages. This development has prompted an interdisciplinary approach to reading research, examining reading through various theoretical frameworks and research methodologies. Moreover, the scope of findings in reading research has extended beyond the frequently studied languages, such as English, German, and Dutch. Current research interest is increasingly focused on less-studied languages, especially those with relatively shallow orthography, rich morphology, flexible word order, and longer word length. As a result, eye-movement corpora have been assembled in numerous languages (e.g., Kennedy et al., 2013; Kliegl et al., 2004; Laurinavichyute et al., 2019; Luke & Christianson, 2018; Pan et al., 2022), as well as for studies on monolingual and bilingual reading (Cop et al., 2017) and cross-linguistic multilingual reading (Kuperman et al., 2023; Siegelman et al., 2022). The primary aim of the present study is to provide empirical data for Persian, a language that has been under-investigated. We specifically follow the methodological scope introduced by Özkan et al. (2021) to choose the set of variables to be included in the analysis.

This expansion, which encompasses less-studied languages with different orthographic and morphological characteristics, is essential for developing a more comprehensive understanding of how language-specific features influence reading behaviors. In this context, Persian, a modern Iranian language, offers an opportunity for investigation. Persian's right-to-left script, rich morphology, and distinct linguistic features make it an ideal candidate for extending the research into eye movement patterns during reading. The following section introduces the Persian Sentence Reading (PSR) Corpus, which aims to facilitate empirical studies on Persian and contribute to the broader cross-linguistic understanding of reading processes.

## Persian as a modern Iranian language

We introduce the PSR Corpus to facilitate further exploration of the influences of unique Persian characteristics on eye movement patterns during sentence reading. Eye movement patterns in reading Latin script languages, which predominantly have a left-to-right reading direction, have attracted substantial attention. Although studies have investigated languages with different reading directions, such as right-to-left (e.g., Yan et al., 2014, for Uighur; Jordan et al., 2014, for Arabic; Pollatsek et al., 1981, for Hebrew) and top-to-bottom (e.g., Su et al., 2020, for Mongolian; Osaka & Oda, 1991, for Japanese), Persian script languages such as Farsi and Dari have remained understudied. This study was designed given the motivation that similar studies on understudied languages would enable comparative analyses between Persian, languages using the same script, such as Arabic, and the ones with different scripts.

The Iranian languages are divided into three historical periods: old, middle, and new. Modern Persian uses the Arabic script, which originated from the Aramaic alphabet. However, four letters** (***p, č, ž*, and *g)* have been added for writing the sounds that are only present in Persian but not in the Arabic language. Persian is an Iranian language that belongs to the West Iranian language family of the Indo-Iranian family within the Indo-European language family (Keshavarz & Ingram, 2002). Three varieties of Persian are recognized: Dari Persian spoken in Afghanistan, Farsi Persian spoken in Iran, and Tajik Persian spoken in Tajikistan, each with dialectal subgroups (Purmohammad et al., 2022). Farsi is the official language of Iran. It is estimated that Farsi is spoken by 110 million speakers worldwide (Dabir-Moghaddam, 2013). In the present study, by Persian, we refer to Farsi.

The modern Iranian languages are divided into “Western” and “Eastern” subgroups. New West Iranian languages are spoken in Iran, and they include Persian (the widest-used Iranian language, and as mentioned above, with Iranian, Afghan, and Tajik variations), Kurdish, Mazandarani, and Gilaki (the two latter spoken across the Caspian Sea in Northern Iran), Lori, Baluchi, Tāti, Tāleši, Lārestāni, Bashkardi, and some others. There are, however, languages spoken beyond this territory and still considered to be West Iranian, like Baluchi (which is also spoken in Pakistan) and Zazaki (spoken in Turkey). Most of these languages do not have a writing system of their own and use the Persian alphabet. The Tajiks, however, make use of the Cyrillic alphabet (Mahmoodi-Bakhtiari, 2004). Therefore, Persian exhibits diglossia, a situation in which two languages or varieties of a language are used under different conditions within a community. Persian has been conceived as a diglossic language as there exists a divergent, highly codified superposed variety besides the primary dialects of the language (Ferguson, 1959, p. 88). Persian also presents differences between the spoken and written forms in all four areas of phonology, semantics, morphology, and syntax, (Mahmoodi-Bakhtiari, 2018a).

As for their grammatical characteristics, the modern Iranian languages do not use markers for case, number, and gender (Mahmoodi-Bakhtiari, 2018b). However, there are markers for the three persons and the two numbers in singular and plural in the New Persian. Persian uses both free lexical morphemes such as *divār* (meaning “wall”) and bound lexical morphemes which constitute the present stems of the verb, such as *mi-rav-am* (incomplete aspect prefix/indicative mood marker-”go” (root)−1sg [first-person singular]. “I go”; Mahmoodi-Bakhtiari, 2018b). Pronominal enclitics have four major functions in Persian. They are used as a possessive marker when attached to a noun phrase: *ketāb* = *etān* (meaning “your book”). They may be attached to prepositions, and act as the object of a preposition: *barāy* = *etān* (meaning “for you”). Pronominal enclitics attach to the transitive verbs and assume the function of the direct object, equivalent to an independent pronoun together with the direct object marker *rā: mi-shenās-am* = *ash* (meaning “I know him”). Finally, there exists a small number of compound verbs in which the object marker is enclitic; however, as the overt subject does not induce agreement on these verbs, they act as the subject of the verb, such as *sard-am ast* (meaning “I feel cold”) (Mahmoodi-Bakhtiari, 2018b).

As of our knowledge at the time of the study, Persian has remained an understudied language in terms of eye movements during reading. Azadfallah et al. (2017) examined the effects of lexical and nonlexical characteristics on eye movement patterns in Persian. They investigated how word length and font type affect eye movement, specifically fixation, saccade, and regression when reading both familiar and unfamiliar Persian passages. The study involved 29 female participants. The stimuli included four text variations: familiar text in lotus font, familiar text in pen font, unfamiliar text in lotus font, and unfamiliar text in pen font. The findings revealed that word length significantly influenced eye movements, specifically fixations and saccades. Moreover, passage type (familiarity or unfamiliarity) significantly affected eye movements, including fixations, saccades, and regressions. However, font type did not show any significant impact on eye movements. Notably, the interaction between word length, font type, and passage type had a significant effect on eye movements.

The current study presents an exploratory analysis and the report of preliminary results of a linear mixed model of single fixation durations as the representative case in the PSR Corpus. By including the neighboring word characteristics, various first-pass eye movement measures, and raw data files, the PSR Corpus also enables theoretical investigations, such as dynamical modulation of perceptual span by foveal processing difficulty (e.g., Kliegl et al., 2006; Özkan et al., 2021), saccadic error explanation of inverted optimal viewing position effect (e.g., Hohenstein et al., 2017), and the investigations of refixation strategies (e.g., Özkan & Acartürk, 2024). In the following sections, we present the methodology of the empirical study, variables included in the PSR Corpus, and our preliminary observations with the results of the linear mixed model of single fixation durations.

## Method

### Participants

Sixty adult native speakers of Persian (*M* = 29.60, *SD* = 4.03, 30 female) with normal or corrected-to-normal vision voluntarily participated in the present study. None of the participants used contact lenses; 16 participants used glasses during the recording sessions. Ten participants were monolingual Persian speakers, while 50 were bilinguals of Azari (a variant spoken in Iran) and Persian. The recording sessions lasted about 30 min. Before the recordings, each participant signed an informed consent form.

A group of 172 participants (native speakers of Persian, *M* = 23.66, *SD* = 5.03, 70 female), participated in a sentential predictability test using the cloze procedure (Taylor, 1953) for the words between the first and the last words of the sentences. The last words were predicted by another group of 15 participants (*M* = 25.83, *SD* = 1.80, 10 female).^1^

### Materials

The experiment stimuli consisted of 99 sentences taken from the Bijankhan corpus (dbrg.ut.ac.ir/Bijankhan/) created by the Database Research Group (at the Faculty of Literature and Human Sciences) at the University of Tehran and funded by the Research Institute for Information and Communication Technology. The corpus consists of 72,708 word types and 2,299,042 word tokens.

The selected sentences respected the following constraints: (1) *Sentence length*. The sentences consisted of nine words at least and 12 words at most (*M* = 10.27, *SD* = 1.01). The sentences were presented on a line each containing 69 characters at most (*M* = 55.76, *SD* = 6.74). (2) *Sentence content/type*. There were no idioms, abbreviations, hyphenated words, numbers, and punctuation marks in the sentences. None of the sentences was a question or an interjective sentence.

### Procedure

**Fig. 1:**
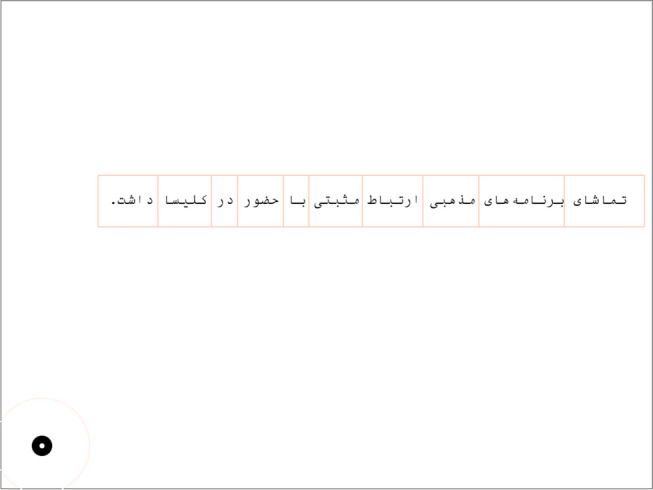
A sample screen presented to the participants. Rectangles around the words and the circle aroundthe fixation marker are interest areas, which were invisible to the participants

Participants read the stimuli sentences in 18 pt. Courier New font on a 24-inch monitor (1024 × 768-pixel resolution) controlled by a 3.6 GHz computer. Participants were seated approximately 66 cm from the eye tracker camera and 73 cm from the screen, each character corresponding to 0.42° of visual angle and approximately 14.03 pixels, horizontally.^2^ Figure 1 illustrates the stimulus presented to the participants, together with interest areas.

Eye movements of the right eye of the participants were recorded with an SR Research EyeLink 1000 Plus desktop mount system with a 1000 Hz sampling rate using the eye-tracker manufacturer software (i.e., Experiment Builder). To avoid head movements and, accordingly, calibration problems, the heads of the participants were positioned on a forehead rest and a chin rest.

**Fig. 2:**
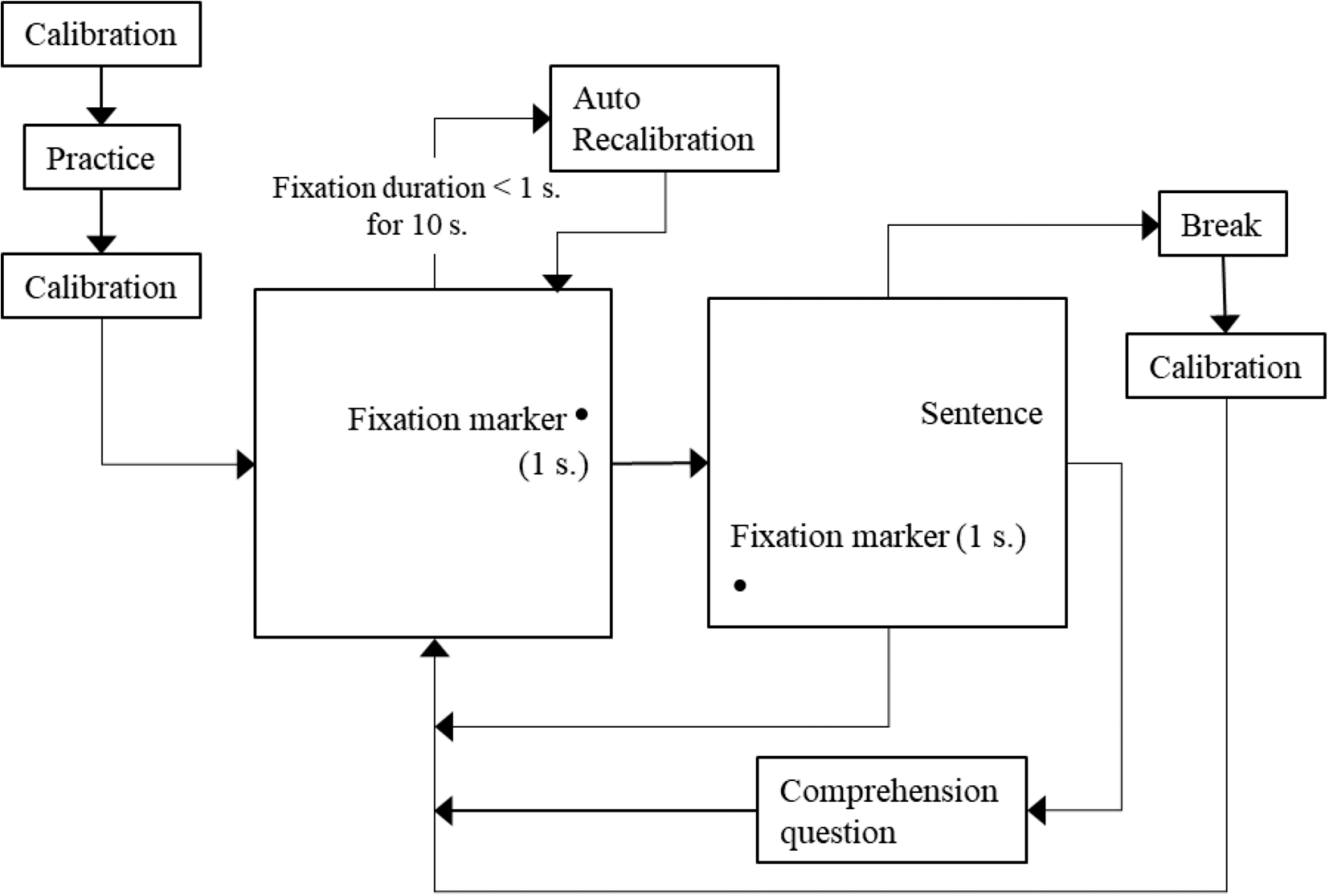
The experimental procedure

The recording session preceded with a practice session (four sentences and four yes/no questions for comprehension) and proceeded with a stimuli set consisting of 99 stimuli sentences and 20 comprehension questions. The session involved five blocks, each presenting the sentences in random order. At the beginning of each session, a standard nine-point grid calibration and validation were used. The calibration was followed by a fixation marker on a blank screen presented prior to each experimental sentence. The marker was placed on the right of the screen at the same coordinates as the first letter of sentences, as Persian has a right-to-left reading direction. The gaze-contingent interest area around the fixation marker served to display the sentence on the screen after 1 s of fixation and send a trigger for the auto-recalibration when needed. The recalibration was triggered if the tracker could not detect a 1-s fixation for 10 s. Another fixation marker, near the left-bottom corner of the screen, allowed the participant to proceed with the next sentence. The auto-recalibration was not linked to the second fixation marker to avoid limiting the reading duration of the participant. Consequently, the sentences were followed either by a yes/no comprehension question, a fixation marker on the right of a blank screen to proceed with the next trial or a break in case of the end of an experiment block. The comprehension questions were related to the previous sentence content to keep the attention of the participants focused on the experiment task. They were presented pseudo-randomly to avoid the systematic expectation of a question.^3^ Participants answered 89% (*SD* = 0.3%) of the questions correctly. Figure 2 illustrates the experimental procedure.

Offline raw data inspection revealed that the average error for the calibration procedure was 0.34, and the average maximum error was 0.72. The system marked four validation results as “FAIR” (mean average error: 0.48) and one as “POOR” (average error: 1.12), which were followed by a successful auto-recalibration procedure.^4^

### Data selection and description

The data from 60 participants were screened for drifts and loss of measurement. Eight trials were excluded from the analyses since the drift correction was not possible by moving fixations up or down or using the drift-correct function of Data Viewer (SR Research, version 3.2.48). One recording session was cancelled due to a technical problem after the participant read 26 sentences. In sum, 325 words from 34 sentences were excluded from the analyses due to technical problems or low data quality. The valid data included for further processing was 60,575 words (99.47% of 60,900 words read by 60 participants).

Following the previous research (Özkan et al., 2021; Rayner, 1998, 2009; Rayner et al., 2012), first-pass eye movement measures were included in the current study. Words were accepted as fixated in the first pass if they received at least one fixation before viewing the following word (i.e., the word on the left for Persian); otherwise, they were accepted as skipped. There were 46,587 words that received at least one fixation (76.5% of valid data) and 13,988 words that were skipped (22.97% of valid data) in the first pass. The complete valid data instances (60,575 words), including skipped instances with annotation, were included in the PSR Corpus.

The words that received the current fixation were labeled as *word n* (i.e., foveal word). Since Persian has a right-to-left reading direction, *word n − 1* indicates the word on the right of word *n*, and *word n* + *1* indicates the word on the left of word *n*. The characteristics of word *n* and its neighbors (i.e., word *n − 1* and word *n* + *1*) were included in the PSR Corpus. Information about PSR is presented in the following section.

### Persian sentence reading corpus

The PSR Corpus^5^ includes the most reported first-pass reading eye movement measures, word characteristics, and predictability norms, in addition to the general variables (Table 1).

**Table 1:** General variables

Code	Variable	Explanation
G1	Participant	The codes that represent participants
G2	Trial index	The order of the trial as presented within the experiment session

### Variables of word characteristics and materials

In the PSR Corpus, the *M#* code next to the variables represents the materials of the study and word characteristics. The features such as code of the sentence and word would be important in a mixed-model analysis as random factors. Word characteristics such as frequency, length, and predictability are provided for a foveal word and its neighboring words to facilitate diverse analyses that could contribute to the discussions related to eye movement control models. The variable codes, variables, and their descriptions are presented in Table 2.

**Table 2:** Variables of word characteristics and materials

Code	Variable	Description
M1	Sentence	The code that represents sentences
M2	Interest area ID	The order of the word within the text, given by the software as is the ordinal ID of the current interest area
M3	Word ID	The ID of the word, indicating the sentence code together with the order of the word in the sentence
M4	Word count	The count of words in the sentence
M5	Word code	The code of each unique word (independent of their order in the sentence)
M6	Word frequency (word *n*, raw)	Word (*n*) frequency as the count of occurrences in the Bijankhan corpus
M7	Word frequency (word *n*, pm)	Word (*n*) frequency as per million
M8	Predictability (word *n*)	The probability of the correct prediction of the word (*n*) (each word predicted by 13 participants)
M9	Word length (word *n*)	Length of word (*n*) as character count
M10	Word frequency (word *n − *1, raw)	Word (*n − 1*) frequency as the count of occurrences in the Bijankhan corpus
M11	Word frequency (word *n − *1, pm)	Word (*n − 1*) frequency as per million
M12	Predictability (word *n − *1)	The probability of the correct prediction of the word (*n − 1*) (each word predicted by 13 participants)
M13	Word length (word *n − *1)	Length of word (*n − 1*) as character count
M14	Word frequency (word *n* + 1, raw)	Word (*n* + *1*) frequency as the count of occurrences in the Bijankhan corpus
M15	Word frequency (word n + 1, pm)	Word (*n* + *1*) frequency as per million
M16	Predictability (word *n* + 1)	The probability of the correct prediction of the word (*n* + *1*) (each word predicted by 13 participants)
M17	Word length (word *n* + 1)	Length of word (*n* + *1*) as character count

### Oculomotor measures

The oculomotor measures were coded as *OM#* in the PSR Corpus. In addition to mostly reported first-pass eye movement measures such as first fixation duration and gaze duration, a reading rate measure (i.e., words per minute) and skipping information was provided. The measures given by the software as pixels (i.e., first landing position and launch site) were converted to letter counts and both versions were included in the PSR Corpus (Table 3).

**Table 3:** Oculomotor measures

Code	Variable	Explanation
OM1	First fixation duration (ms)	The duration of the first fixation on the word in the first pass, without taking into account whether an interest area with a higher ID was fixated before, given by the software
OM2	Gaze duration (ms)	The sum of the fixation durations on the word in the first pass (gaze duration), without taking into account whether an interest area with a higher ID was fixated before, given by the software
OM3	Fixation count	The count of the fixations on the word in the first pass. Values given by the software without taking into account whether an interest area with a higher ID was fixated before were corrected manually to account for skipped instances (i.e., fixation count for skipped instances were set to *0*)
OM4	First landing position (pixels)	The first landing position on the word—i.e., the difference between the X coordinate of the first fixation and the X coordinate of the lower-right corner of the interest area of the word. In pixels, given by the software
OM5	Launch site (pixels)	The distance of the fixation preceding the first fixation to the word (launch site). In pixels, given by the software
OM6	First fixation location (letters)	Location of the first fixation on the word in terms of character count
OM7	Launch site (letters)	Launch site in terms of character count
OM8	Incoming saccade amplitude (letters)	Incoming saccade amplitude in terms of character count
OM9	Outgoing saccade amplitude (letters)	Outgoing saccade amplitude in terms of character count
OM10	Words per minute	Reading rate as words read per minute
OM11	Skipped word	Whether the word was skipped; *1* indicating skipped words. In addition to instances that were marked as *1* by the software (i.e., if the interest area was not fixated at all, or fixated after a fixation on an interest area with a higher ID), instances that have a first fixation on the space that succeeds the word were also marked as *1* manually

### Further analyses

Depending on the researchers’ analytical focus, there may be a need for further elimination of the data with different criteria which cannot be extracted from the provided eye movement measures. To account for such cases, a variable set with the *E#* code was provided in the PSR Corpus (Table 4).

**Table 4:** Variables for further analyses and elimination

Code	Variable	Explanation
E1	First and last words	The first words and last words of the sentences; *1* indicating the first or the last word
E2	Regressive eye movements	Words that have a regressive first fixation or regressive outgoing saccade; *1* indicating a regressive eye movement
E3	Fixation on space	Whether the fixation preceding the first fixation or the fixation succeeding the last fixation was on the space between words; *1* indicating a fixation on the space
E4	Previous and next fixation not on a word	Whether the fixation preceding the first fixation or the fixation succeeding the last fixation was on a word; *1* indicating that the mentioned fixation was not on a word

## Preliminary observations

Among the valid words included in the corpus, 52.92% received a single fixation, 24.99% received multiple fixations, and 23.09% were skipped in the first-pass reading. Words that received multiple fixations were longer, less frequent, and less predictable than those that received a single fixation and those that were skipped. On the other hand, high-frequency, short, and more predictable words tended to be skipped. Relative fixation locations were closer to the center of the words if they received a single fixation. If the words received multiple fixations, on the other hand, the relative first fixation locations were closer to the beginning of the words. Incoming saccade amplitudes were approximately seven characters. However, approximately four characters of outgoing saccade amplitudes were less relative to reports of other languages.

A further investigation of data revealed that the low values of outgoing saccade amplitude were due to regressive eye movements which had negative outgoing saccade values. After eliminating regressive eye movements (14.61% of valid data), the mean outgoing saccade amplitude was approximately seven characters (*M* = 7.25, *SD* = 3.6). These findings are largely compatible with the previous studies (Rayner, 1998, 2009; Rayner et al., 2012, for reviews). Table 5 lists means and standard deviations of eye movement measures and word characteristics over valid words included in the PSR Corpus.

**Table 5:** Means and standard deviations (in parenthesis) for oculomotor variables and word characteristics broken by fixation counts

Variables			Valid instances	Single fixation cases	Multiple fixation cases	Skipped cases
Number of words			60,575	31,450	15,137	13,988
Number of fixations			1.4	1.00	2.24	-
			(0.68)	(NA)	(0.61)	
Fixation duration (ms)	First fixation		226.18	233.08	211.83	-
			(102.76)	(106.56)	(92.74)	
	Sum of fixations (gaze)		308.19	233.08	464.0	
			(189.27)	(106.56)	(224.94)	
First fixation location (character count)	Relative to first letter		2.78	2.95	2.42	-
			(1.44)	(1.38)	(1.48)	
	Relative to word center		− 0.02	0.04	− 0.14	
			(0.24)	(0.23)	(0.23)	
Amplitude (character count)	Incoming saccade		6.47	6.54	6.28	-
			(1.96)	(1.99)	(1.84)	
	Outgoing saccade		4.15	4.1	4.25	
			(10.3)	(10.38)	(10.13)	
	Launch site		3.73	3.61	4.05	
			(2.08)	(2.1)	(1.99)	
Word frequency (pm)	Word *n*		2185.01	2915.34	667.6	18,035.72
			(6738.11)	(7886.47)	(2664.51)	(17,161.01)
	Word *n − 1*		6745.33	6258.24	7992.74	3204.72
			(13,401.16)	(12,912.06)	(14,505.3)	(9282.07)
	Word *n* + *1*		7082.03	6770.58	7724.73	3786.09
			(13,201.08)	(12,829.08)	(13,915.84)	(9796.34)
Predictability (probability)	Word *n*		0.15	0.18	0.11	0.26
			(0.28)	(0.3)	(0.23)	(0.36)
	Word *n − 1*		0.13	0.13	0.12	0.11
			(0.23)	(0.23)	(0.22)	(0.21)
	Word *n* + *1*		0.21	0.22	0.19	0.14
			(0.32)	(0.33)	(0.29)	(0.27)
Word length (character count)	Word *n*		4.94	4.51	5.84	2.68
			(1.76)	(1.51)	(1.91)	(1.17)
	Word *n − 1*		4.34	4.42	4.15	4.68
			(1.98)	(1.97)	(1.99)	(1.8)
	Word *n* + *1*		4.24	4.25	4.22	4.65
			(1.83)	(1.82)	(1.86)	(1.96)

### Single fixation duration model

As being the representative case (51.92% of valid words included in the PSR Corpus), single fixation durations (SFDs) were modeled with a linear mixed model using the lmer() function of the lme4 package (version 1.1–3; Bates et al., 2015b) in the R environment (version 4.3.3, 64-bit build; R Core Team, 2024). We applied the same procedure used in Özkan et al. (2021), based on Kliegl (2007). The analysis in the current study was exploratory. The model was constructed in an incremental fashion starting with a base model retrieved from Kliegl (2007, p. 532, Table 1). The random structure was visually inspected using the lattice package (version 0.21.8; Sarkar, 2008) to detect the relevant random slopes and investigate the word as a random factor. While adding new random factors/slopes to the model according to visual inspections, if the model converged at each step, the model with a new component was tested against the simpler one using the anova() function. The model selection $\alpha$-level of likelihood ratio test was 0.2 (i.e., the *p*-level for model comparison was set to 0.2) following Matuschek et al. (2017). Whenever the anova() result indicated that the new component contributed to the model, the random structure of the more complex model was tested using the rePCA() function of the RePsychLing package (version 0.0.4; Baayen et al., 2015). The summary of the rePCA() function output provides the cumulative proportion of variance explained by random slopes of each random factor. However, the result does not indicate which of the random slopes was not supported by data. The bottom-up random structure construction approach used in the current study enabled us to evaluate if including a random slope in the model resulted in a too complex random structure to be supported by data (i.e., the last two components accounted for 100% of the variance, meaning that one of them was not necessary). In other words, if the inclusion of the new random slope did not cause the model complexity related to the random structure to exceed the principle component count cumulatively accounting for 100% of the variance, the more complex model was selected, or otherwise, the simpler model was retained (Bates et al., 2015a). As a result, word as random factor (${\chi }^{2}\left(1\right)$ = 708.65, *p* < 0.2), length of words *n* and *n* − 1 (WL0 and WL1, respectively), and outgoing saccade amplitude (OSA) as random slopes of participant (WL0: ${\chi }^{2}\left(1\right)$ = 4.63, *p* < 0.2; WL1: ${\chi }^{2}\left(1\right)$ = 11.90, *p* < 0.2; OSA: ${\chi }^{2}\left(1\right)$ = 54.21, *p* < 0.2) contributed to the model while satisfying the mentioned selection criteria. 

The relationship between covariates and SFD was visually inspected using the languageR package (version 1.5.0; Baayen, 2019) to consider nonlinear relationships. Whenever the visual inspection showed a nonlinear relationship, the covariate was added to the model as a nonlinear component and tested against the simpler model with the same procedure used for the random structure. The model that included the quadratic component of length of word *n* + *1* (WF2: ${\chi }^{2}\left(1\right)$ = 20.70, *p* < 0.2) was better with the model selection $\alpha$-level of likelihood ratio test of 0.2 (Matuschek et al., 2017). The variance inflation factors (VIFs) were calculated to test for multicollinearity (Lefcheck, 2012). There were no fixed factors that had a VIF value greater than 4. Accordingly, the model was not simplified after this stage (Zuur et al., 2010). The final model was reported with the *p* values using the lmerTest package (version 3.1–3; Kuznetsova et al., 2017). Line and 95% confidence band are partial effects for figures, retrieved from LMM estimates by using the remef package (version 1.0.7; Hohenstein & Kliegl, 2023). Graphs were constructed using the ggplot2 package (version 3.5.0; Wickham, 2016).^6^ In Table 6, the linear mixed model parameter estimates for the final model of single fixation durations (SFD) are presented.

**Table 6:** Linear mixed model parameter estimates for single fixation durations

Fixed effects:	Estimate	*SE*	*df*	*t* value	Pr( >|*t*|)
(Intercept)	5.42	0.017	87.04	314.43	< 0.01
Predictability *n* (P0)*	− 0.010	0.003	171.51	− 3.33	< 0.01
Predictability *n − 1* (P1)	− 0.006	0.002	1983.74	− 0.36	0.72
Predictability *n* + *1* (P2)*	0.01	0.002	1531.04	5.18	< 0.001
Word length *n* (WL0)	0.043	0.066	472.00	0.65	0.52
Word length *n − 1* (WL1)*	0.165	0.030	596.31	5.42	< 0.001
Word length *n* + *1* (WL2)	− 0.059	0.035	1374.30	− 1.68	< 0.1
Relative fixation location (RFL)*	− 0.144	0.022	66.08	− 6.53	< 0.001
RFL (quadr.-IOVP curvature)*	− 0.467	0.063	17,044.47	− 7.39	< 0.001
Incoming saccade amplitude (ISA)*	0.122	0.006	18,023.88	19.71	< 0.001
Outgoing saccade amplitude (OSA)*	− 0.042	0.012	82.42	− 3.59	< 0.001
Word frequency *n* (WF0)*	− 0.064	0.007	340.31	− 9.36	< 0.001
Word frequency *n − 1* (WF1)*	− 0.026	0.004	1471.29	− 6.27	< 0.001
Word frequency *n* + *1* (WF2)*	0.014	0.005	1374.69	3.01	< 0.01
WF2 (quadr.)*	0.011	0.002	1551.73	4.56	< 0.001
WF0:WL0*	− 0.09	0.04	460.75	− 2.27	< 0.05
WF0:WF1*	0.01	0.003	2290.99	4.07	< 0.001
WF0:WF2	0.001	0.003	1601.51	0.21	0.83
WL0:WF2*	0.071	0.028	3063.11	2.52	< 0.05
WL0:P2	0.01	0.018	5370.88	− 0.55	0.59
Random effects:					
Groups	Name	Variance	SD		
word	(Intercept)	0.008	0.09		
sentence	(Intercept)	0.0014	0.04		
Participant	(Intercept)	0.0144	0.12		
Participant	P0	0.0002	0.01		
Participant	WL0	0.0113	0.11		
Participant	RFL	0.0178	0.13		
Participant	WF0	0.0005	0.02		
Participant	WL1	0.0062	0.08		
Participant	OSA	0.0046	0.07		
Residual		0.0708	0.27		

Number of obs: 18,874, groups: word, 532; sentence, 99; participant, 60

Annotation: * indicates that the effect of the variable is significant; a colon between variables indicates interaction

P: predictability; WL: word length; RFL: relative fixation location; OSA: outgoing saccade amplitude; WF: word frequency

The results are largely compatible with previous findings (Rayner, 1998, 2009; Rayner et al., 2012, for reviews). A comparison between the common factors of the current analysis and that of a similar analysis on German using the Potsdam Sentence Corpus revealed remarkable similarities (cf. Kliegl, 2007). Model estimates showed that the directions of the relationship between word characteristics and single fixation duration were largely similar to those in Kliegl (2007). The only differences were observed in the coefficients of the length of the next word (positive in Kliegl, 2007) and the frequency of the next word (negative in Kliegl, 2007). Similarly, the significance status of the coefficients was similar in the two studies, except for the length of the foveal word and the predictability of the previous word (both were significant in Kliegl, 2007). Besides, the significance status of the interactions was also the same, except for the interaction between the length of the foveal word and the predictability of the next word (it was significant in Kliegl, 2007). The relationship between viewing position (i.e., relative fixation location and saccade amplitudes) and single fixation duration was the same in the two studies regarding the significance of the effects. The signs of the coefficients of fixation location (both linear and quadratic) and incoming saccade amplitude were also the same in both studies. However, the coefficient of outgoing saccade amplitude in Kliegl (2007) was positive. Below is a more detailed report of the results of the current analysis.

**Fig. 3:**
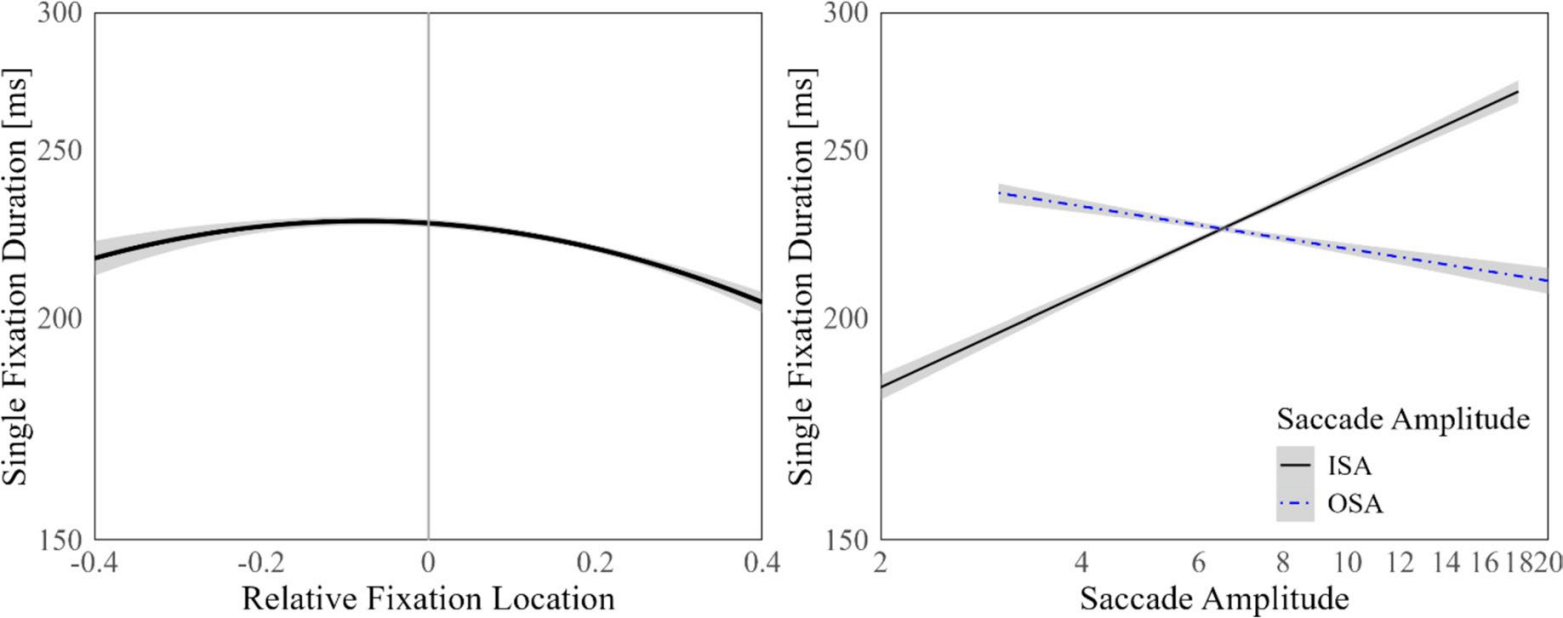
Oculomotor measures and SFD. A zero value of RFL indicates the center of the word (the grey horizontal line), negative values indicate the first half (right of the word center in Persian) of the word, and positive values indicate the second half (left of the word center in Persian)

A frequently reported effect, namely, the inverted optimal viewing position effect (i.e., IOVP) was observed (Vitu et al., 2001; e.g., Nuthmann et al., 2005; Yan et al., 2014, Hyönä et al., 2018; Özkan et al., 2021). In other words, significant effects of the linear and quadratic components of RFL indicated that SFD was longer when the fixation was located closer to the center of the word when it was located closer to the edges. As the distance from the previous fixation increased (i.e., increasing incoming saccade amplitude [ISA]) SFD values tended to increase as well. On the other hand, outgoing saccade amplitude (OSA) influenced SFD negatively. The relationship between SFD and oculomotor measures included in LMM is illustrated in Fig. 3.

The predictability of word *n* (P0) had a negative effect on SFD. In other words, the less predictable a word, the longer the fixation duration, in line with previous research (Rayner, 1998, 2009; Rayner et al., 2012, for reviews). While the effect of the predictability of word *n − 1* (P1) on SFD was not significant, we observed a significant positive effect of the predictability of word *n* + *1* (P2) on SFD. Although controversial, the positive effect of P2 can be construed as evidence for a memory retrieval process for highly predictable upcoming words while fixating on foveal words (cf. Kliegl et al., 2006).

The decreasing effects of the frequencies of word *n* and word *n − 1* (i.e., WF0 and WF1) on SFD were significant. As expected, more frequent words received shorter SFDs. The frequency of word *n* + *1* (WF2), on the other hand, had a quadratic influence on SFD in line with the raw data inspections. The negative effect of WF2 on SFD for WF2 values below approximately two on the logarithmic scale became a positive effect for values greater than that WF2 value. Among word length covariates included in the LMM, only the effect of the length of word *n − 1* (WL1) on SFD was significant. The significant influences of word characteristics on SFD are illustrated in Fig. 2.

**Fig. 4:**
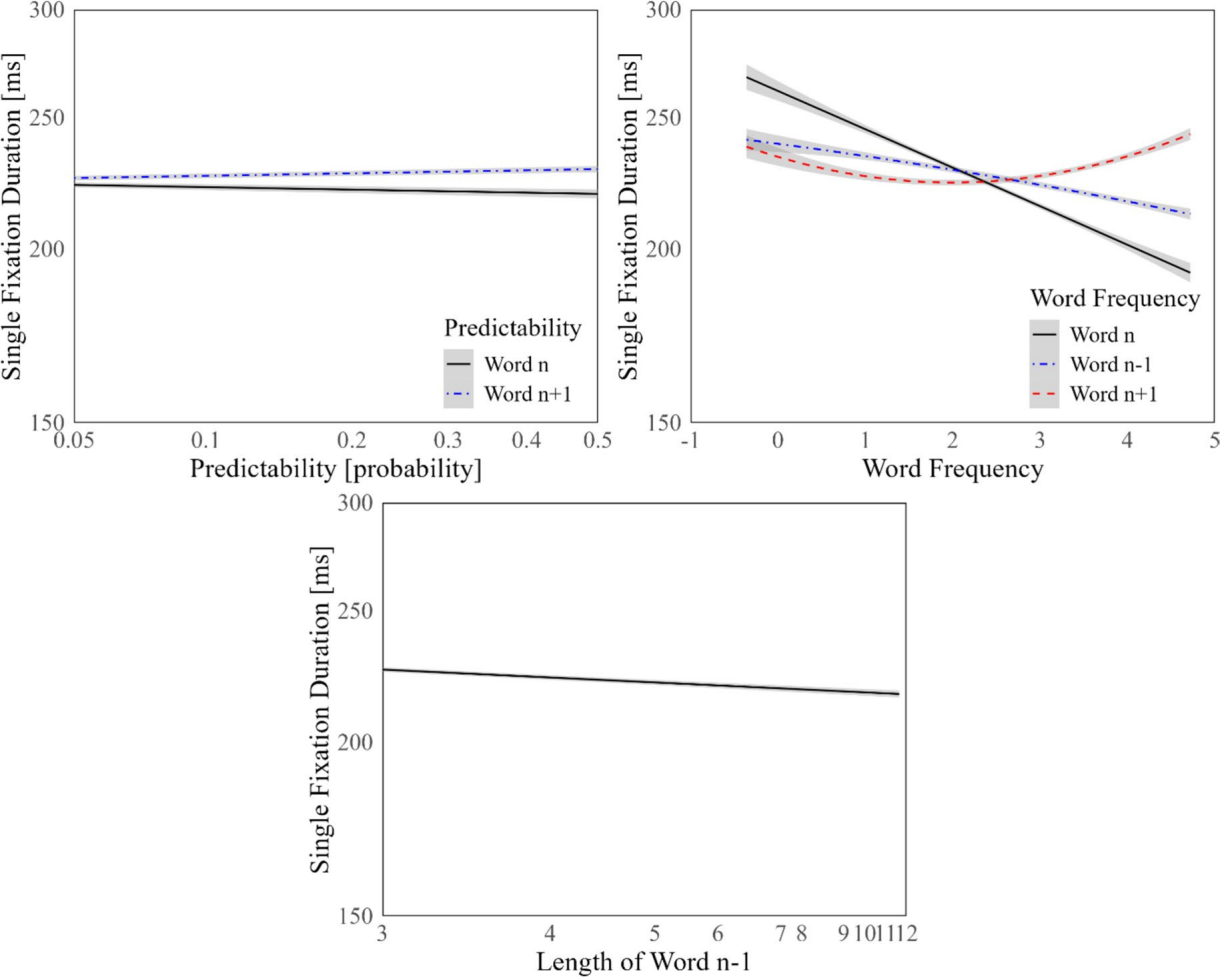
Word characteristics and SFD. Units of word frequency on the *x*-axis are log-10 power of words per million

**Fig. 5:**
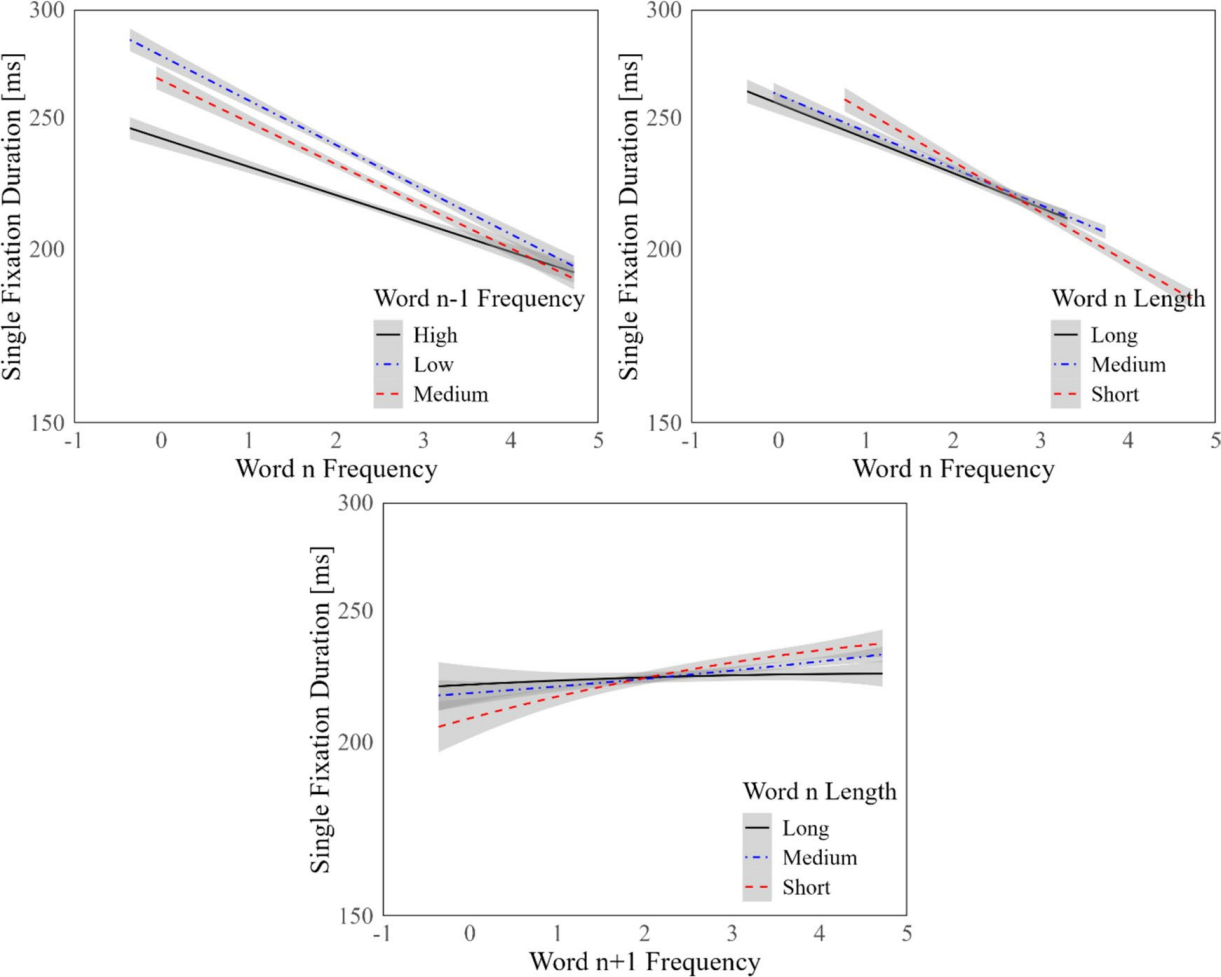
Interaction effects. Units of word frequency on the *x*-axis are log-10 power of words per million. WF1 values are grouped as such: high WF1 values are greater than three on the logarithmic scale (> 1000 pm), low WF1 values are less than two on the logarithmic scale (< 100 pm), and medium WF1 values are in between. WL0 values are grouped as such: long words have more than 5 letters, short words have fewer than 4 letters, and medium words have in between

There were five interaction terms in the LMM: the interaction of the frequency and length of word *n* (WF0 and WL0), the interaction of the frequencies of word *n* and word *n − 1* (WF0 and WF1), the interaction of the length of word *n* and frequency of word *n* + *1* (WL0 and WF2), the interaction of the frequencies of word *n* and word *n* + *1* (WF0 and WF2), and the length of word n and the predictability of word *n* + *1* (WL0 and P2). The first three of these interaction terms were significant, while the last two interaction terms were not significant. The negative but not significant effect of WL0 and the negative effect of WF1 on SFD was lost among high WF0 values. The positive influence of WF2 on SFD among short foveal words was almost completely lost among long foveal words (Figs. 4 and 5).

## Conclusion

We presented a set of eye movement measures recorded during reading Persian sentences, establishing a corpus of Persian sentence reading (PSR Corpus). The variables in the corpus, the methodology behind it, the data selection procedure, and preliminary findings are reported. Together with the descriptive statistics of lexical characteristics of foveal and neighboring words and mostly reported eye movement measures, a linear mixed model of single fixation durations as the representative case of the PSR Corpus is provided. We observed that the results were in line with the robust findings in the literature, including the mean fixation durations and saccade lengths, the negative influence of word frequencies on fixation durations, and the inverted optimal viewing position effect, to name a few. Furthermore, a comparison between the results reported in the current study and that of a similar study using the Potsdam Sentence Corpus revealed remarkable similarities regarding the effects of word characteristics and viewing position on single fixation duration (Kliegl, 2007). Persian, as an understudied language, has similarities and differences with languages studied previously such as the script and language family. The exploratory analysis presented in the current study did not consider the unique characteristics of Persian. Further analyses that focus on those would reveal the relationship between Persian-specific characteristics and eye movement measures. We believe that providing Persian reading eye movement patterns will contribute to the investigation of the interplay between eye movements during reading and language characteristics.

## Data Availability

The files that include raw data files, datasets, stimulus sentences, variable explanations, and the files related to LMM analysis can be downloaded from the Persian Sentence Reading Corpus (PSR Corpus) in the Open Science Framework (OSF) Repository, https://osf.io/4w362/. The PSR main dataset is provided as a Comma-Separated Values file, PSRC.csv. The variable explanations are in an Excel file, PSRC_Variables.xlsx. The stimulus sentences are provided in a Portable Document Format, PSRC_stimuli.pdf. The detailed explanations of the linear mixed model of single fixation duration (i.e., LMM construction process, covariate explanations, detailed results, and R codes) are combined in one Hypertext Markup Language file, Persian_SFD_LMM.html, and provided together with the data used in LMM analysis in Persian_SFD_LMM.zip. Data and materials are available at the Open Science Framework OSF Repository, https://osf.io/4w362/.

## Footnotes

1 The resulting predictions are from 13 participants for each word included in the aggregated data. An additional file that includes all correct prediction counts and number of predictions was also added to the Persian Sentence Reading Corpus (PSR Corpus) in the Open Science Framework OSF Repository, https://osf.io/4w362/.

2 The maximum character allowed on a line with Courier New font, 18 pt, left margin 28 px, and right margin 42 px on a 1024 × 768 resolution screen was 68. As Courier New font is monospaced, with each character covering the same amount of area, each character corresponded to 14.0294 px horizontally (rounded to 14.03 px in calculations). The information was used to calculate the visual angle for each character and fixation distances in letter count. The pixel counterparts of the fixation distances are also included in the dataset.

3 The presentation order of the questions to each participant was the same. Each randomly presented sentence had a corresponding comprehension question. They were presented after the sentences as follows: the first, third, and fifth blocks: the third, fourth, tenth, and 15th sentences in the blocks; the second block: the first, fifth, ninth, and 18th sentences; the fourth block: the second, seventh, eighth, and 14th sentences. In other words, only the order of question presentation was fixed, and the sentences and their corresponding questions were presented randomly. Therefore, to give an example, each participant answered a question after the third trial of the first block, but the randomly selected sentence (and hence the question) for that trial was different for different participants.

4 A file that includes validation results extracted from ASCII files, together with the extraction codes and ASCII files, is available under the PSRC_data_files folder in Persian Sentence Reading Corpus (PSR Corpus) in the Open Science Framework OSF Repository, https://osf.io/4w362/

5 The data and materials used in the analyses were from the unpublished thesis work of Soleymani (2018) with a re-inspection of the data.

6 The details of the model construction process and results are available in the Persian Sentence Reading Corpus (PSR Corpus) in Open Science Framework OSF Repository, https://osf.io/4w362/

## Code availability

Codes are available at the Open Science Framework OSF Repository, https://osf.io/4w362/.
